# Barriers, knowledge, and training related to pharmacists’ counselling on dietary and herbal supplements: a systematic review of qualitative studies

**DOI:** 10.1186/s12913-021-06502-4

**Published:** 2021-05-25

**Authors:** Jeremy Y. Ng, Umair Tahir, Simran Dhaliwal

**Affiliations:** Department of Health Research Methods, Evidence, and Impact, Faculty of Health Sciences, McMaster University, Michael G. DeGroote Centre for Learning and Discovery, Room 2112, 1280 Main Street West, Hamilton, ON L8S 4K1 Canada

**Keywords:** Dietary supplement, Herbal, Pharmacist, Pharmacy, Qualitative research, Qualitative systematic review

## Abstract

**Background:**

Pharmacists are recognized as one of the most accessible healthcare providers and are licensed to advise patients on drugs and health products including dietary and herbal supplements (DHSs). The objective of this study was to identify barriers, knowledge, and training that pharmacists report related to DHSs counselling.

**Methods:**

MEDLINE, EMBASE, AMED and CINAHL were systematically searched from database inception to May 8th, 2020. Eligible articles contained qualitative data with a specific focus on pharmacists’ perceived knowledge, training, and barriers to DHSs counselling. Relevant data were extracted, and a thematic analysis was conducted.

**Results:**

Nineteen articles met the inclusion criteria. The following three main themes were identified: challenges to pharmacists obtaining DHSs education, postgraduate workplace challenges surrounding DHSs, and pharmacists’ perceived role and importance on DHSs. Low knowledge of DHSs and the limited regulations surrounding DHSs acting as a barrier to counselling were common findings supported by the eligible articles.

**Conclusions:**

A lack of pharmacists’ knowledge and awareness of DHSs stems from a variety of factors including a lack of education and training in the field, limited regulations surrounding DHSs, and inadequate availability of DHS information resources in the pharmacy. Pharmacists were unable to confidently counsel patients due to these aforementioned factors in addition to reporting that they lacked time. Further research that reviews pharmacy education and workplace training, and improving DHS regulations are warranted future directions.

## Background

The use of dietary and herbal supplements (DHSs) is highly prevalent worldwide. In countries including the United States, United Kingdom, and Denmark, it has been found that 35–60% of adults use DHSs [1, 2]. In 2016, the Natural Health Product Survey among Consumers conducted in Canada found that 56% of respondents had taken DHSs such as vitamins and minerals at least once per week [3]. From the same survey, 56% of the respondents considered themselves to have poor knowledge about the safety of DHSs [3]. In certain clinic populations, the prevalence of use is even higher; almost all (99%) patients used DHSs, with 46% using DHSs concurrently with prescription medications in a large Canadian naturopathic medicine clinic population, however, 42% of the DHS users had not disclosed this information to their primary care provider [4]. In 1999, a survey conducted in the United States identified that 53% of DHS users believed that DHSs were completely safe to use and rarely ever caused harm [5]. As DHSs are available without a prescription, consumers may self-medicate and suffer the potential risks of drug-DHS interactions. According to a study led by the Mayo Clinic in 2002–2003, 34% of DHS users were at risk of suffering potential herb-drug interactions [5]. A number of studies have identified DHS-drug interactions leading to adverse events [6, 7], which is of great concern as many patients often do not consult with their healthcare provider(s) before using DHSs.

The regulatory environment related to DHSs varies across many different countries. Two categories of regulations exist for DHSs: premarket and postmarket regulations [8–10]. Effective premarket regulations require evidence on safety and efficacy to be provided through scientific literature [8]. Additionally, the manufacturing process, quality, and labelling of ingredients must uphold good standards as set by regulatory authorities in each country [8]. Postmarket DHSs regulations usually consist of market surveillance surrounding the products’ quality, efficacy, safety, and adverse events as reported by consumers [8]. If a DHS is found to be adulterated, misbranded, or harmful to consumer health, regulatory authorities will take appropriate action and may remove the product from the market [8]. Ideally, DHSs have both premarket and postmarket regulations, as is the case in countries such as Canada [11], while in countries such as the United States, only the latter exists [12]. In countries such as New Zealand, the United Kingdom, Japan, and Australia, only DHSs with therapeutic claims must adhere to both premarket and postmarket regulations, while those with non-therapeutic claims are only assessed through postmarket regulations [13–16].

Regardless of how DHSs are regulated, pharmacists are in a unique position to educate patients about the use, efficacy, side effects, and potential interactions with prescription medications associated with such products. An Australian survey found that 87–92% of consumers expected pharmacists to be able to provide adequate and reliable information about the safety and efficacy of DHSs [17]. Furthermore, a systematic review identified that pharmacists believe that they have the professional responsibility to counsel patients on the safe use of DHSs [18], and in some jurisdictions, pharmacists are already providing recommendations about DHSs to their patients frequently. A survey found that 40% of US pharmacists and 28.6% of UK pharmacists recommended multivitamins more than five times a week [19].

Knowing that pharmacists frequently offer DHSs advice to patients for a wide range of health conditions, it is critical for pharmacists to have a reasonable degree of knowledge about these products. Despite this necessity, multiple surveys have indicated that pharmacists have low knowledge of and confidence in the safety and efficacy of DHSs and drug-DHSs interactions [20, 21]. A preliminary literature search on this topic suggested that a need existed to explore the complexity of pharmacists’ experiences with DHSs and assess the context in which findings related to their practice in this area are situated. Therefore, the objective of this review was to identify the barriers, knowledge, and training that pharmacists report related to counselling on DHSs.

## Methods

### Approach

A qualitative systematic review was conducted to examine pharmacists’ knowledge of DHSs using standard methods [22] and Preferred Reporting Items for Systematic Reviews and Meta-Analyses (PRISMA) criteria [23]. A qualitative systematic review seeks to combine findings across multiple studies to understand the complexities of individuals’ experiences and perceptions, rather than assessing the effectiveness of an intervention [24, 25]. A protocol was not registered.

### Eligibility criteria

Eligible studies reported qualitative data (e.g. qualitative surveys, interviews, focus groups, and observational studies) on pharmacists’ perceptions of their knowledge, training, and barriers related to counselling on DHSs. Mixed-methods studies that contained a qualitative component were also eligible, however, studies that contained solely quantitative data were ineligible. For the purpose of this study, we defined DHSs to include vitamins and minerals, herbal remedies, homeopathic medicines, traditional medicines such as traditional Chinese medicines, probiotics, amino acids, and essential fatty acids, which was based on published definitions of DHSs, similar products, or their synonyms [26–29]. Only English-language articles were included, and all studies not meeting the aforementioned eligibility criteria were excluded.

### Searching and screening

MEDLINE, EMBASE, AMED and CINAHL were searched on May 11th, 2020 from inception to May 8th, 2020. JYN designed the search strategy **(**Table 1**)** to encompass both keywords and indexed headings related to DHSs, pharmacists, and qualitative research methods. UT and SD independently screened the titles and abstracts recovered from all four databases, independently and in duplicate. Any discrepancies were resolved through discussion between all three authors. The same process was undertaken for the full-text screening of eligible studies.

**Table 1:** MEDLINE Search Strategy for Studies Reporting Qualitative Data Regarding the Pharmacists’ Knowledge of DHSs Executed May 11, 2020

Database: Ovid MEDLINE(R) and Epub Ahead of Print, In-Process & Other Non-Indexed Citations, Daily and Versions(R) < 1946 to May 08, 2020> Search Strategy: -------------------------------------------------------------------------------- 1 dietary supplement*.mp. (67505) 2 exp. Dietary Supplements/ (75009) 3 natural health product*.mp. (415) 4 natural product*.mp. (37584) 5 vitamin*.mp. (266966) 6 exp. Vitamins/ (320422) 7 mineral*.mp. (190102) 8 exp. Minerals/ (164674) 9 (herb* adj1 (medic* or therap* or supplement*)).mp. (24484) 10 exp. Medicine, East Asian Traditional/ or exp. Medicine, Chinese Traditional/ or exp. Herbal Medicine/ or exp. Plants, Medicinal/ or exp. Phytotherapy/ (108689) 11 tcm.mp. (10185) 12 exp. Drugs, Chinese Herbal/ (42957) 13 traditional Chinese medicine.mp. (19492) 14 herbalism.mp. (149) 15 exp. Homeopathy/ (4722) 16 homeopath*.mp. (6503) 17 probiotic.mp. (16394) 18 exp. Probiotics/ (16825) 19 or/1–18 (961675) 20 (pharmaci* or pharmacy).mp. (88138) 21 exp. Pharmacy Research/ or exp. Education, Pharmacy, Continuing/ or exp. Pharmacy/ or exp. Evidence-Based Pharmacy Practice/ or exp. Clinical Pharmacy Information Systems/ or exp. Pharmacy Residencies/ or exp. Education, Pharmacy/ or exp. Community Pharmacy Services/ or exp. Education, Pharmacy, Graduate/ or exp. Pharmacy Service, Hospital/ (30644) 22 exp. Pharmacists/ (16527) 23 or/20–22 (88138) 24 (qualitative* or survey* or focus group* or interview* or observational* or descriptive* or mixed method*).mp. (1736863) 25 19 and 23 and 24 (602) 26 limit 25 to english language (536) ***************************	

### Data extraction

A data extraction form was created a priori to collect information from each eligible article. The following items were extracted and summarized: title of study; author; year published; country/countries of participants; term(s) used to describe DHS; regulatory status of the DHSs; types of DHSs included in the study; study methodologies; theoretical underpinnings; pharmacist type; number of participants; outcomes; themes discussed; main findings; challenges encountered by the study population; limitations to the study; and study conclusions. UT and SD independently extracted data and discrepancies were resolved through discussion with JYN.

### Thematic analysis

Thematic analysis was conducted based on the data extracted from Table 2 and Table 3, which included the themes, main findings, challenges encountered, limitations and conclusions. The available qualitative data was first summarized in tables and then analyzed by all three authors. UT and SD interpreted the evidence from the included papers to identify common key concepts. JYN, UT, and SD used words and phrases that captured key concepts to create codes, thematically organized codes into groups, and presented a discussion based on the research question as well as highlighted knowledge gaps in the currently published literature. Any disagreements were resolved through discussion.

**Table 2:** General Characteristics of Eligible Studies

Title	Author and Year Published	Country/Countries of Participants	Term(s) Used to Describe Dietary and Herbal Supplements (DHS)	1. Regulatory Status of the DHSs Included in the Study 2. If Applicable, How are the DHSs Included in the Study Regulated?	Types of DHS Included in Study	Methodologies Used	Theoretical Underpinning	Types of Pharmacists Included in Study	Number of Participants	Outcomes of Study	Themes Discussed
Community pharmacists’ professional practices for complementary medicines: a qualitative study in New Zealand	Barnes and Butler. 2020 [30]	New Zealand	Complementary Medicines (CMs)	1. Yes, but limited 2. Medicines Act regulations, Dietary Supplement Regulations, proposed Therapeutic Products Act (which will replace the current Medicines Act in New Zealand)	Herbals, homoeopathics, vitamins, minerals, traditional medicine, and “natural” or plant-based products	Semi-structured interviews	Not reported	Community	27 participants: all pharmacists	Pharmacists’ views on CMs in New Zealand, motivations and justifications for selling CMs, and professional and ethical issues CMs raise	1. Description of CMs 2. Pharmacists’ personal perspectives 3. Motivations for stocking and selling CMs 4. Community pharmacists’ advisory role for CMs 5. Knowledge, training and information sources on CMs 6. Professional and ethical issues 7. Role of pharmacy organizations
Integrating traditional Chinese medicines into professional community pharmacy practice in China-Key stakeholder perspectives	Yao et al. 2020 [31]	China	Traditional Chinese Medicine (TCM) and Herbal Medicines (HM)	1. Yes, but limited 2. Not reported	Not reported	Semi-structured interviews	Grounded Theory	Community and clinical	14 participants: 7 pharmacists, 1 regulatory authority representative, 2 pharmacy school representatives, 1 professional organization representative, 2 chain pharmacy representatives and 1 key opinion leader	Perceptions of key stakeholders about the enablers and challenges to pharmacists’ adopting a role in patient care associated with the concurrent use of HMs	1. Purposes of HMs use by the public 2. Perception about integrative medicine 3. Perception about the safety of HMs 4. Perception about pharmacists’ role in HMs 5. Major barriers hindering pharmacists taking up a more professional role related to HMs 6. Actions needed to support pharmacists taking up a more professional role related to HMs
Advancing the pharmacist’s role in promoting the appropriate and safe use of dietary supplements	Harnett et al. 2019 [32]	United States	Dietary Supplements (DS) and Natural Products (NP)	1. Yes 2. Regulated by the FDA and FTC	Herbal products, nutritional and vitamin and mineral supplements, homoeopathic preparations	Semi-structured interviews	Grounded Theory	Practicing pharmacists in various settings (including retail and health systems) and pharmacist officers/leaders in various professional and regulatory organizations	22 participants: 12 pharmacists and 10 key stakeholders	Key stakeholder and pharmacist perceptions about the actions needed to enable pharmacists to fulfill a professional role related to DS use	1. Education and training 2. Strategies for ensuring high standards related to regulation of DS safety and quality assurance 3. Workplace resources 4. DS research
Barriers to pharmacists adopting professional responsibilities that support the appropriate and safe use of dietary supplements in the United States: Perspectives of key stakeholders	Ung et al. 2019 [33]	United States	Dietary supplements (DS)	1. Yes, but limited 2. FDA	Vitamins, minerals, herbals, nutritional supplements including probiotics and fish oils	Semi-structured key informant telephone interviews	Grounded theory	Community	22 participants: 12 pharmacists and 10 stakeholders	Pharmacists’ and key stakeholders’ perceptions and opinions about accepting roles that ensure the appropriate and safe use of DS	1. Awareness of DS use and knowledge of potential benefits and harms 2. Perceived responsibility of pharmacists in caring for patients regarding DS use 3. Incorporation of DS use into pharmacists’ current practice 4. Participants’ responses to proposed responsibilities related to DS
Informing the homeopathic practice for Turkish pharmacists: reviewing the example of Portuguese community pharmacies	Cavaco et al. 2017 [34]	Turkey	Homeopathy	1. Yes 2. Turkish Pharmacy Law and Turkish Traditional and Complementary Medicine Act	Homeopathic remedies	Semi- structured interviews and an initial documentary analysis	Not reported	Community	6 participants: all pharmacists	Portuguese pharmacists’ attitudes (knowledge, feelings, and behaviour) towards pharmacy-based homeopathy	1. General homeopathic practice 2. Emerging feelings 3.Healthcare market opportunity 4.Homeopathic education 5. Regulatory framework 6. Patients’ support
Development of a strategic model for integrating complementary medicines into professional pharmacy practice	Ung et al. 2017 [35]	Australia	Traditional Medicine (TM) and Complementary Medicine (CM)	1. Yes, but limited 2. Therapeutic Goods Administration (TGA)	Herbal medicine, dietary supplement, health supplement, vitamins, minerals, and natural products	Focus group interviews (FGIs)	Not reported	Community	11 participants: all pharmacists	Pharmacists’ perspective on how barriers to the integration of TM/CM products into pharmacy practice could be resolved.	1.Dilemmas that pharmacists had to face during their day-to-day practice **Four major next steps:** 1. Education and training2. Build CM evidence base3. CM information resources4.Workplace support for best CM practice
Key stakeholder perspectives on the barriers and solutions to pharmacy practice towards complementary medicines: an Australian experience	Ung et al. 2017 [36]	Australia	Traditional Medicine (TM) and Complementary Medicine (CM)	1. Yes 2. Therapeutic Goods Administration (TGA)	Dietary supplements, herbal medicines, health supplements, vitamins, minerals and natural products, traditional medicine	Semi-structured interviews	Not reported	Community	11 participants: 2 pharmacists, 1 pharmacy owner, and 8 key stakeholders	Pharmacists and key stakeholder leaders’ perceptions and opinions regarding the barriers that hinder pharmacists from providing care related to the use of CMs and solutions that would support pharmacists’ in extending their role in this area.	**9 barriers hindering pharmacists’ duty of care regarding CMs:** 1. Insufficient knowledge about CMs 2. Pharmacists’ attitude towards CMs 3. Lack of research skills 4. Lack of evidence for efficacy and safety of CM 5. Lack of access to trustworthy information and support 6. Lack of time 7. Consumers’ attitudes 8. Lack of a defined role for pharmacists 9. Poor inter-professional communication with doctors **7 solutions to support pharmacists’ extended role in CMs:** 1. The integration of CMs into pharmacists’ undergraduate and professional education development 2. A clear definition of the pharmacists’ role in CMs 3. Pharmacies employing a naturopath 4. The establishment of reliable, easily accessed information 5. Promoting quality CMs research 6. Collaboration among health care professionals 7. The provision of consumer education
Assessing the Awareness and Knowledge on the Use of Probiotics by Healthcare Professionals in Nigeria	Amarauche 2016 [37]	Nigeria	Probiotics	1. Yes 2. National Agency for Food and Drug Administration and Control (NAFDAC).	Not reported	Semi-structured questionnaires	Not reported	Community	221 participants: physicians, pharmacists, dentists, and nurses *Did not specify how many of each.	The knowledge and awareness of healthcare professionals in Nigeria on probiotics	Not reported
Perceptions of traditional, complementary and alternative medicine among conventional healthcare practitioners in Accra, Ghana: Implications for integrative healthcare	Kretchy et al. 2016 [38]	Ghana	Traditional complementary and alternative medicine (TM-CAM) products	1. Yes, but limited 2. Traditional and Alternate Medicines Directorate (TAMD)	Not reported	Semi-structured interviews	Not reported	Hospital	23 participants: 5 physicians, 8 pharmacists, 5 nurses and 5 dieticians	Perceptions of conventional healthcare professionals on integrative medicine	1. Knowledge gap 2. The paradox of TM/CAM 3. Experience of use and prescription 4. Guided integration
Pharmacists’ knowledge and attitudes about natural health products: a mixed-methods study	Kheir et al. 2014 [39]	Qatar	Natural health products (NHP) & Complementary and alternative medicine (CAM)	1. Yes, but limited 2. Not reported	Herbal products (Echinacea, Saw palmetto, St John’s wort, Valerian, Cranberry Extract, Black cohosh, Ginseng, Ginger, *Ginkgo biloba*, Garlic), Vitamin supplements, Traditional Chinese medicine, Homeopathic products	Mixed methods: Questionnaire and focus-group (FG) discussions	Not reported	Community and hospital	110 participants: 110 pharmacists (92 pharmacists from quantitative component and 18 from qualitative component)	Attitude and knowledge of pharmacists in Qatar towards natural health products	1. NHP Knowledge 2. Perception of CAM 3. Reference Sources 4. Challenges and Barriers 5. NHP Regulations
Community pharmacists’ attitudes relating to patients’ use of health products in Japan	Asahina et al. 2012 [40]	Japan	Health products	1. Not reported 2. N/A	Not reported	Focus group interviews (FGIs)	Not reported	Community	16 participants: pharmacists	Japanese pharmacists’ attitudes relating to patients’ use of health products	1. Pharmacists’ ideas on health products 2. Perceived barriers to communication with patients about health products 3. Perceived facilitators to communication with patients about health products 4. Need for support in information provision and monitoring of patients
Understanding pharmacists’ experiences with advice-giving in the community pharmacy setting: a focus group study	Simmons-Yon et al. 2012 [41]	United States	Complementary and Alternative Medicines (CAM)	1. No 2. N/A	Not reported	Focus group interviews (FGIs)	Not reported	Community	31 participants: 10 community pharmacists and 21 advanced doctor of pharmacy students	The experiences of community pharmacists providing advice about symptoms and CAM	1. Pharmacists as advisors 2. Pharmacists as medical liaisons 3. CAM-related advice 4. Educational needs
Assessment of herbal weight loss supplement counseling provided to patients by pharmacists and nonpharmacists in community settings	Jordan et al. 2011 [42]	United States	Herbal dietary supplements	1. Not reported 2.N/A	Apple Cider Vinegar Diet, Chitosan, CitraMax, CLA (conjugated linoleic acid), CLA Extreme,CLA-1300, Ephedra, Ezee Slimming Patch, Fücothin, Green tea/green tea extract (various brands), Less Stress Weight Control, Hoodia (various brands),Hydroxycut; Hydroxycut Hardcore, Mega-T Green Tea, Metabolife, Metabolift, Optim-3 CLAS Supreme,Sea Thin, Slender Formula,SlenderWeigh, Slim 7, Thermonex, Thermo Slim Tea, Trim Spa, Ultimate Fat Metabolizer, Ultra Plan Metabolic Supreme Herbal	Interviews	Not reported	Community	52 participants: 27 pharmacists and 25 nonpharmacists (cashiers, sales clerks, pharmacy technicians)	The amount of appropriate counselling provided to patients by nonpharmacists and pharmacists in retail settings regarding herbal dietary supplements for weight loss	1. Herbal supplements and lactation/pregnancy 2. Supplement-drug interactions 3. Effects of herbal dietary supplements for weight loss 4. Safety of herbal dietary supplements for weight loss 5. Recommended herbal product 6. Potential adverse effects of herbal supplements for weight loss 7. Potential allergens in herbal supplements for weight loss
Reporting natural health product related adverse drug reactions: is it the pharmacist’s responsibility?	Walji et al. 2011 [43]	Canada	Natural health products (NHPs)	1. Yes 2. Canadian regulations state requirements for the manufacture, packaging, labelling, storage, importation, distribution, and sale of NHPs.	Herbal and homeopathic medicines, vitamins and minerals, probiotics, amino acids and essential fatty acids	Semi- structured interviews	Ethnomethodology	Community	12 participants: all pharmacists	Pharmacists’ experiences with and responses to receiving or identifying reports of suspected ADRs associated with NHPs from pharmacy customers	1.Responsibility 2. Perception of competency 3. Workplace challenges 4. Pharmacists’ professional role
Over-the-counter advice seeking about complementary and alternative medicines (CAM) in community pharmacies and health shops: an ethnographic study	Cramer et al. 2010 [44]	United Kingdom	Complementary and alternative medicine (CAM)	1. Yes, but limited 2. Not reported	Homoeopathic products, anthroposophical remedies, flower remedies, herbs, food supplements (e.g. spirulina, *Aloe vera*, probiotics and enzymes) and single vitamins and minerals (e.g. vitamin C and zinc)	Semi-structured interviews and non-participant observation	Ethnography	Community	54 participants: pharmacists, owners/managers and counter assistants (*n* = 24) and 30 customers	Customer advice seeking about complementary and alternative medicine	1. Help with diagnosis 2. Help finding a general remedy 3. Help with a specific product 4. Free advice 5. Pastoral care 6. Just buying
Responding to patient demand: community pharmacists and herbal and nutritional products for children	Robinson et al. 2010 [45]	England	Herbal and nutritional products (HNPs) and Complementary and alternative medicine (CAM)	1. Not reported 2. N/A	Homeopathic products, herbs, vitamins, minerals, and food supplements	Semi structured interviews and diary recordings (diary sheet), questionnaires	Not reported	Community	22 participants: 5 pharmacists and 17 pharmacy staff	The attitudes and behaviour of pharmacists working in a multi-ethnic community regarding herbal and nutritional products (HNPs) for children	Not reported
An evaluation of pharmacist and health food store retailer’s knowledge regarding potential drug interactions associated with St. John’s wort	Sim et al. 2010 [46]	Canada	Natural health product (NHP)	1. Not reported 2. N/A	St. John’s wort and echinacea	Interview outline (questionnaire) and interview	Not reported	Community	30 participants: 24 pharmacists and 6 NHP retailers	Pharmacists and natural health product retailer’s knowledge on St Johns wort	Not reported
Exploratory study of factors influencing practice of pharmacists in Australia and Thailand with respect to dietary supplements and complementary medicines	Kanjanarach et al. 2006 [47]	Australia and Thailand	Dietary supplements and complementary medicines (DS/CM)	1. Yes - Australia; Minimally regulated -Thailand 2. Not reported	Not reported	Semi-structured interviews	Not reported	Community and hospital	20 participants: all pharmacists	The opinions and practices of pharmacists in Australia and Thailand in relation to dietary supplements and complementary medicines (DS/CM)	1. How DS/CM have been used 2. Factors influencing customers’ purchasing of DS/CM 3. Efficacy, safety, and necessity of use of DS/CM 4. Attending customers buying or seeking advice 5. Evaluating the necessity and safety of use of DS/CM 6. Providing information about DS/CM 7. Assisting customers in product selection and criteria to select DS/CM products for customers 8. Criteria for selection of DS/CM products to sell in a pharmacy 9. Impact of DS/CM on pharmacy business
An investigation of pharmacists’ and health food store employees’ knowledge about and attitudes toward kava	Webb et al. 2004 [48]	Canada	Herbal medication	1. No (stated that Kava is unregulated) 2. N/A	Kava	Interviews	Not reported	Community	58 participants: 28 pharmacists and 30 health food store employees	The information provided to potential clients by pharmacists and health food retailers regarding the herbal medication kava	Not reported

*Abbreviations:* complementary and alternative medicine (CAM); complementary medicine (CM); dietary supplements (DS); dietary supplements and complementary medicines (DS/CM); food and drug administration (FDA); focus group (FG); focus group interviews (FGIs); federal trade commission (FTC); herbal medicine (HM); herbal and nutritional products (HNPs); national agency for food and drug administration and control (NAFDAC); natural health products (NHP); natural product (NP); traditional and alternate medicines directorate (TAMD); traditional complementary and alternative medicine (TM-CAM); traditional complementary medicine (TCM); therapeutic goods administration (TGA); traditional medicine (TM)

**Table 3:** Outcomes and Findings of Eligible Studies

Title	Author and Year Published	Main Findings	Challenges Encountered by Study Population	Limitations to the Study	Conclusions
Community pharmacists’ professional practices for complementary medicines: a qualitative study in New Zealand	Barnes and Butler. 2020 [30]	1. Participants found it difficult to clearly describe products they considered complementary medicines. 2. Perspectives towards CMs ranged from strongly supportive to somewhat sceptical 3. Consumer demand and profits were the most stated motivations for selling CMs. 4. Pharmacists limited knowledge/training and lack of evidence of efficacy were the most common ethical issues surrounding CMs that pharmacists acknowledged. 5. Very few pharmacists explicitly referred to the CMs-related statements in the Pharmacy Council of New Zealand’s Code of Ethics to guide their practice.	1. Lack of knowledge/training on CMs 2. Lack of specific regulatory framework for CMs 3. Lack of evidence-based studies	1. Non-representative, purposive/convenience sample: pharmacists supportive of CMs may be over-represented 2. Possibility of social desirability bias	Pharmacists justify selling CMs despite their lack of knowledge in these products. Pharmacists are mindful of professional and ethical issues regarding CMs, but their practice is not guided by the Pharmacy Council of New Zealand’s Code of Ethics. There lacks a specific regulatory framework for CMs in New Zealand.
Integrating traditional Chinese medicines into professional community pharmacy practice in China-Key stakeholder perspectives	Yao et al. 2020 [31]	1. Participants agreed that pharmacists should play a role in drug safety associated with concurrent use of TCM and western medicine. 2. Barriers exist within the government, education, pharmacy, pharmacist, and research sectors. 3. Prominent themes were a lack of clarity in defining the pharmacists’ role surrounding HMs and a disconnect between current regulatory standards and education system. 4. The most important enablers identified were the development of policies that support pharmacy practice and practice guidelines, the review of competency standards, and registration criteria.	1. Lack of the competence to provide professional service 2. Lack of knowledge about Chinese medicines or conventional medicines 3. Lack of professional image 4. Lack of motivation or interests in direct patient care 5. Lack of information source 6. Lack of practice guidelines	1. Small sample size only representative of a small number of key stakeholders and certain regions of the country 2. The exclusion of other ethnic minority medicine 3. The perspectives of patients and consumers were not included	Key stakeholders report that there are gaps in pharmacists’ knowledge in HMs and therefore are unable to provide comprehensive pharmaceutical care. Guiding principles that outline standards for such use would serve as a baseline for professional expectations and as a framework to model pharmacy education.
Advancing the pharmacist’s role in promoting the appropriate and safe use of dietary supplements	Harnett et al. 2019 [32]	1. Pharmacists proposed they could develop and promote themselves in DS. 2. Four key areas surrounding DS identified by participants as needing improvement were (1) Education and training; (2) Strategies for ensuring high standards related to DS safety and quality assurance (3) Workplace resources (4) DS Research.	1. Lack of education and training 2. Lack of regulations 3. Lack of workplace resources 4. Lack of DS research	1. Small sample size limits extrapolation of the results.	Pharmacists and key stakeholders hold reasonable ideas on how to overcome the challenges facing pharmacy related DS use. Pharmacists believe that quality education and training on DS, as well as improvement of DS regulation on a governmental and industry level are appropriate next steps.
Barriers to pharmacists adopting professional responsibilities that support the appropriate and safe use of dietary supplements in the United States: Perspectives of key stakeholders	Ung et al. 2019 [33]	1. Pharmacists acknowledge their ethical and professional responsibilities regarding dietary supplements. 2. Due to multiple barriers, most pharmacists are not expecting to assume responsibilities regarding dietary supplements soon.	1. Perceived lack of an evidence base and support to access information 2. Concerns about the regulation of DS 3. Services for DS were not considered by pharmacists to be within their scope of practice 4. Barriers in being able to include DS in electronic records 5. Many pharmacists did not believe they should accept responsibility to report suspected adverse events 6. Ambiguity in expectations; lack of clarity	1. The qualitative approach found rich information not likely to be gleaned from a quantitative survey, precluding generalizability to a larger group 2. Limited number of interviewee participants precludes extrapolation of the results 3. The interviewees comprised 2 separate groups; however, the design precludes direct comparisons as well as any sort of consensus building	Pharmacists lack in their understanding of their professional roles and responsibilities surrounding dietary supplement awareness and use. Key stakeholders need to be involved to improve the situation.
Informing the homeopathic practice for Turkish pharmacists: reviewing the example of Portuguese community pharmacies	Cavaco et al. 2017 [34]	1. Pharmacists’ attitudes regarding homeopathy is greatly influenced by their education and relationship with homeopathic physicians. 2. Specialized homeopathic education was considered an important factor for success.	1. Lack in knowledge of homeopathy and the regulatory framework that surrounds it 2. Lack of reliable information resources	Not reported	Pharmacists believe that job satisfaction and appropriate legal frameworks are important features of homeopathic practice. Specialized homeopathic education and improved patient counselling were also considered by pharmacists to be important factors for success. Further investigation is required to examine how the present qualitative findings can be generalized to a larger sample of pharmacists.
Development of a strategic model for integrating complementary medicines into professional pharmacy practice	Ung et al. 2017 [35]	1. Pharmacists identified 7 key CMs related dilemmas that they face during their day-to-day practice. 2. Four developments were proposed that require a collaborative effort: education and training; building the evidence base; developing reliable and accessible information resources; and workplace support for best practice	1. Dilemmas surrounding TM/CM 2. Lack of education 3. Lack of access to reliable information resources	1. An over-representation of pharmacists working in the independent pharmacies and under-representation of community pharmacists from urban areas. 2. Snowballing and self-selection of participants	This study proposed a strategic model to integrate TM/CM products into pharmacy practice. Pharmacists suggest the need for improvement in education and the regulatory policies related to CMs.
Key stakeholder perspectives on the barriers and solutions to pharmacy practice towards complementary medicines: an Australian experience	Ung et al. 2017 [36]	1. The main barriers identified by many pharmacists were a lack of CMs knowledge, doubts about the evidence-base, a lack of research skills and access to reliable information 2. Participants proposed the integration of CMs curricula into under-graduate and professional pharmacy education and defining a clearer role for pharmacists’ standard of practice. 3. Participants had apposing opinions about the role of naturopaths in pharmacies.	1. Insufficient knowledge about CMs 2. Pharmacists’ attitude towards CMs 3. Lack of research skills 4. Lack of evidence for efficacy and safety of CM 5. Lack of access to trustworthy information and support 6. Lack of time 7. Consumers’ attitudes 8. Lack of a defined role for pharmacists 9. Poor inter-professional communication with doctors	1. The findings are only representative of Australia 2. Small sample size	Many barriers exist preventing pharmacists from properly fulfilling their responsibilities surrounding CMs. Collaboration between stakeholders is required to plan the implementation of this extended role for pharmacists.
Assessing the Awareness and Knowledge on the Use of Probiotics by Healthcare Professionals in Nigeria	Amarauche 2016 [36]	1.Healthcare professionals in Nigeria possess limited knowledge and awareness of probiotic products. 2. Amongst physicians, pharmacists, dentists and nurses, pharmacists were the most knowledgeable on probiotics.	1. Lack of knowledge 2. Limited access to information on probiotics	Not reported	Healthcare professionals in Nigeria have limited awareness and knowledge of the use of probiotics. Seminars and workshops should be hosted to target this issue.
Perceptions of traditional, complementary and alternative medicine among conventional healthcare practitioners in Accra, Ghana: Implications for integrative healthcare	Kretchy et al. 2016 [38]	1. Participants were worried about the possible negative effects of TM-CAM. 2. Participants’ knowledge of TM-CAM was low. 3. Participants recognized alternative medicine to be as important as conventional medicine.	1. Pharmacists’ lack of confidence in the use of TM-CAM products	1. Results are generalizable since a qualitative approach was adopted 2. Participants were based in more affluent areas, where demand for TM-CAM may generally be different from more deprived areas	Conventional healthcare professionals welcome the idea of integrative medicine. Practitioners’ knowledge on the safety and regulation of TM-CAM must be improved.
Pharmacists’ knowledge and attitudes about natural health products: a mixed-methods study	Kheir et al. 2014 [39]	1. Most pharmacists had average to poor knowledge about NHPs. 2. Poor access to evidence-based information limited pharmacists’ abilities to counsel patients. 3. Pharmacists criticized undergraduate pharmacy education for inadequate preparation to deal with NHPs. 4. Pharmacists deem natural health products to be safe.	1. Lack of education on NHPs 2. Ethical dilemmas - Large profit margin and financial bonus associated with the sale of NHPs presents a sense of pressure on pharmacists to sell NHPs	1. Only Qatar-based pharmacists with contact addresses with the College of Pharmacy were contacted, limiting the generalizability of the results 2. Poor response rate	Pharmacists are disadvantaged in providing adequate services surrounding CAM due to a lack of knowledge and limited access to information resources. A continuing pharmacy education program would help pharmacists improve their NHP related knowledge.
Community pharmacists’ attitudes relating to patients’ use of health products in Japan	Asahina et al. 2012 [40]	1. Pharmacists were not comfortable inquiring about patients’ use of health products due to the lack of scientific evidence on safety and efficacy and feared they could not advise patients properly or answer their questions. 2. Pharmacists expressed their concern regarding the ambiguity surrounding their professional role as a pharmacist. 3. Pharmacists who facilitated discussions with patients regarding natural health products were motivated by their duties as a pharmacist to keep patients healthy and safe.	1. Communication between healthcare professionals on CAM 2. Lack of scientific evidence 3. The ambiguity surrounding pharmacists’ role in relation to health products	1. Focus group interviews cause a lack of generalizability of the results 2. Deficiencies in heterogeneity within the focus groups, especially with respect to sex distribution 3. The number of participants and the number of FGIs were limited due to the small number of available participants 4. The differences between users and nonusers of i-PHISS were not explored in detail	Ambiguity regarding pharmacists’ professional role surrounding health products may cause a lack of communication with patients. Pharmacists must realize the objectives of counselling patients interested in natural health products. Further research is needed to clarify the roles of pharmacists and to ensure the implementation of appropriate educational objectives in pharmacy curriculums.
Understanding pharmacists’ experiences with advice-giving in the community pharmacy setting: a focus group study	Simmons-Yon et al. 2012 [41]	1. Patients try to delay physician visits by seeking pharmacist advice. 2. Most pharmacists felt uncomfortable recommending CAM because of the lack of evidence and regulation. 3. To prepare pharmacy graduates for employment in community settings, participants suggested that pharmacy curricula expand training on symptom triage, pharmacist–patient communication, and CAM.	1. Lack of education 2. Lack of evidence 3. Lack of regulations	1. The perspectives of the focus group participants may not be representative of pharmacists and students in other regions 2. Most of the focus group participants were pharmacy students 3. The results are based on self-reported data from the participants 4. Most of the participants worked in chain pharmacies	A lack of education, evidence and regulations on CAM reduces pharmacists’ comfort levels in recommending CAM to patients. Pharmacy education should be enhanced to include training on CAM use. Pharmacies should provide easily accessible educational materials for pharmacists on CAM products to better enable them to answer patient inquiries.
Assessment of herbal weight loss supplement counseling provided to patients by pharmacists and nonpharmacists in community settings	Jordan et al. 2011 [42]	1. Pharmacist and nonpharmacist responses to questions regarding product safety differed significantly. 2. Most pharmacists and nonpharmacists advised against the use of herbal products during pregnancy and breast-feeding. 3. When unsure about safety, nonpharmacists did not usually refer the investigators to a health care provider. 4. Pharmacists usually referred the investigator to a health care provider when unsure about safety. 5. Patients may not be given complete or correct information about herb–drug and herb–disease interactions.	1. Lack of knowledge 2. Lack of time 3. Lack of reliable information 4. Lack of training	1. Exclusion of participants outside of Phoenix, AZ 2. The interview process did not allow for interviewers to obtain a detailed history of each participant’s training in herbal products	Counselling on herbal supplements provided by personnel at health food stores, retail stores, grocery stores, and pharmacies was highly variable. The herbal and medical communities should work together to create mechanisms by which patients can be properly informed about herbal products.
Reporting natural health product related adverse drug reactions: is it the pharmacist’s responsibility?	Walji et al. 2011 [43]	1. Pharmacists generally did not submit reports of adverse events associated with NHPs to the national ADR reporting system due to several barriers. 2. Pharmacists who reported adverse events did so because they saw themselves as ‘knowledge generators’ 3. There is a need for more formal education and training on NHPs.	1. Lack of time 2. Complexity of reporting process 3. Lack of knowledge 4. Varying opinions in pharmacists’ responsibilities and roles in ADR reporting	1. Small sample size not representative of all of Ontario or Canadian pharmacists	Pharmacists’ perceptions of their professional roles may explain their reporting behaviour for suspected adverse drug reactions associated with NHPs. Information regarding safety and ADRs is lacking in herbal medicines. The improvement of pharmacy education and clearly defining the professional role of a pharmacist are important next steps.
Over-the-counter advice seeking about complementary and alternative medicines (CAM) in community pharmacies and health shops: an ethnographic study	Cramer et al. 2010 [44]	1. Most customers purchasing CAM products need extensive help selecting an appropriate remedy 2. Pharmacists (and counter staff) lack training on CAM products, preventing them from providing appropriate and detailed advice. 3. Health shops may lack knowledge regarding potential interactions between pharmaceutical products and CAM.	1. Lack of proper training and education	Not reported	Customers require help selecting CAM products since there are a wide variety of products offered in pharmacies and health stores. Pharmacists and health product retailers are lacking in the support needed surrounding CAM. Staff in health food stores and pharmacists would benefit from further training and education.
Responding to patient demand: community pharmacists and herbal and nutritional products for children	Robinson et al. 2010 [45]	1. Pharmacists were generally open to herbal and nutritional products and were keen to meet the increasing demand. 2. Pharmacists reported feeling competent to give advice on HNPs. 3. Pharmacists wished to increase their knowledge on HNPs to maintain professionalism, but information on HNPs was limited. 4. Pharmacists understand and empathize with customer demand for HNPs.	1. Lack of knowledge	1. Small sample size and specific location; findings from this study may not be generalizable	Pharmacists acknowledge and empathize with customer demand for herbal and nutritional products, but they may require continued professional training.
An evaluation of pharmacist and health food store retailer’s knowledge regarding potential drug interactions associated with St. John’s wort	Sim et al. 2010 [46]	1. Most pharmacists and NHP retailers recognized that St. John’s wort could be useful for improving mood. 2. Overall low knowledge on potential herb-drug interactions.	1. Insufficient education and training on NHPs 2. Lack of quick access to necessary information about herbal medications	1. Small sample size included only community-based pharmacists 2. Incorrect interpretations of responses may have occurred due to the complexity in answers provided by respondents	Pharmacists and natural health product retailers must become more educated about potential herb-drug interactions and have access to resources to evaluate potential threats.
Exploratory study of factors influencing practice of pharmacists in Australia and Thailand with respect to dietary supplements and complementary medicines	Kanjanarach et al. 2006 [47]	1. Australian pharmacists evaluated the safety of DS/CM in the same way as for conventional medicines. 2. Thai pharmacists quickly assessed customers’ health before selling them DS/CM products. 3. Both Thai and Australian pharmacists provided inadequate information about DS/CM products and their efficacy. 4. Australian pharmacists recommended products that they were knowledgeable about. 5. Thai pharmacists would suggest brands that yielded a higher profit margin. 6. Neither Australian nor Thai pharmacists were proactive in providing services to support the use of DS/CM.	Not reported	1. The interviewer was a pharmacist, which could have influenced the way in which respondents answered questions 2. The possible impact of transcription and translation on the meaning of messages	Thai nor Australian pharmacists took initiative to provide appropriate services to ensure DS/CM products were being used correctly. Australian and Thai pharmacists had different motives to sell or recommend DS/CM products to customers. Pharmacists’ professional role in relation to DS/CM products should be clarified.
An investigation of pharmacists’ and health food store employees’ knowledge about and attitudes toward kava	Webb et al. 2004 [48]	1. Both pharmacists and health food store employees agreed on similar conditions that could be treated with kava. 2. Most pharmacists were cautious about the safety of Kava. 3. Health food employees were likely to deny the possibility of adverse effects associated with kava use. 4. There was inconsistency in the level of caution advised by both groups regarding safety and efficacy of kava. 5. Pharmacists and retailers were greatly misinformed about kava.	Not reported	Not reported	Pharmacists and health food store employees have different views on the safety of Kava. There is a significant level of misinformation provided by health food store employees about Kava compared to pharmacists. Misinformation could lead to negative health consequences and a waste of customers’ money.

*Abbreviations:* adverse drug reactions (ADRs); complementary and alternative medicine (CAM); complementary medicine (CM); dietary supplements (DS); dietary supplements and complementary medicines (DS/CM); focus group (FG); focus group interviews (FGIs); herbal medicine (HM); herbal and nutritional products (HNPs); natural health products (NHP); natural product (NP); traditional complementary and alternative medicine (TM-CAM); traditional complementary medicine (TCM); traditional medicine (TM)

## Results

### Search results

**Fig. 1:**
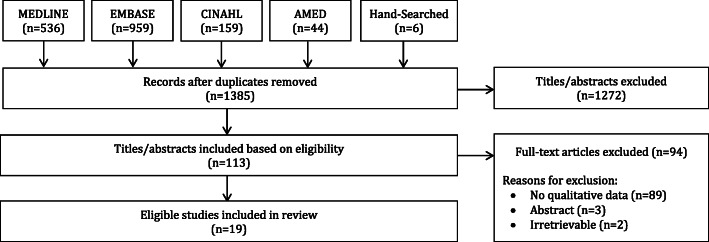
PRISMA Diagram

Searches identified a total of 1695 items, of which 1385 were unique, and 1272 titles and abstracts were eliminated, leaving 113 full-text articles for further review. Of those, 94 were deemed ineligible, because they did not include qualitative data (*n* = 89), were conference abstracts (*n* = 3) or were irretrievable (*n* = 2), leaving a total of 19 articles that were included in this qualitative systematic review [30–48]. A PRISMA diagram can be found in Fig. 1.

### Characteristics of included studies

As shown in Table 2, eligible articles were published from 2004 to 2020 and originated from 12 countries. The largest number of studies originated from the United States (*n* = 4). Most studies’ pharmacists practiced in a jurisdiction where regulations on DHSs existed but were limited (*n* = 7). Although many terms were used to describe dietary and herbal supplements (DHSs), the most common included natural health products (*n* = 3), dietary supplements (*n* = 3), and complementary and alternative medicine (*n* = 3). The most common research method used across eligible articles was the qualitative interview (*n* = 28). Most studies’ participants consisted of only community pharmacists (*n* = 15). The details associated with all eligible article characteristics can be found in Table 2.

### Findings from thematic analysis

In total, three main themes emerged from our analysis and are described below.

### Theme 1: challenges to DHS education

Many studies found that pharmacists’ education fell short of providing them with a thorough understanding of DHSs [30, 31, 33, 34, 37, 39, 41–43]. While some pharmacists expressed that they lacked a DHS education component in their pharmacy curricula [32, 35, 43], those that did receive training stated that they were not confident about their DHS knowledge [42, 43]. Furthermore, the pharmacists suggested that recent graduates may have less knowledge on this topic due to reduced DHS content in the curriculum [42, 43]. Pharmacists described that they were conscious of their poor knowledge and awareness of DHSs, including their safety, efficacy, and drug-DHS interactions [30, 35–38, 43, 46]. Others reported that much of their limited knowledge came from word-of-mouth and the media [38]. This lack of DHSs knowledge decreased pharmacists’ willingness to counsel patients on certain products, such as probiotics [37–40, 42, 43, 45]. Many pharmacists agreed with the need for continuing education surrounding DHSs and suggested more DHS curriculum should be incorporated into their university training [30, 32, 34, 35, 37, 40, 45].

### Theme 2: post-graduation workplace challenges

#### Sub-theme 2.1: DHS training and resources in the workplace

Following graduation, pharmacists also expressed that DHS training in the workplace was inadequate, which was a factor that added to their lack of DHS knowledge [36, 38, 40, 44]. One study noted that many of the pharmacists interviewed had received no formal training on DHSs in the pharmacy and had been introduced to only some herbal medicines [38]. Pharmacists have expressed their desire for increased DHS training in order to gain a comprehensive understanding of these products and to provide better counselling to inquiring patients [31, 38, 40]. Additionally, the lack of reliable information about DHSs was a key contributing factor to pharmacists’ hesitation to counsel patients interested in DHSs [31, 35, 39, 40]. Pharmacists stated that they used evidence-based information to assess the risk-benefits of pharmaceutical medications and similarly require such information for DHSs, particularly with regards to safety and efficacy, in order to effectively counsel their patients [30, 33, 36, 41, 46, 48]. Some also indicated that because of the lack of information, their knowledge about such resources came from media sources, including advertisements [38]. To combat this lack of training, some pharmacists suggested the development of a readily accessible online database for providing information on DHSs [32, 36].

#### Sub-theme 2.2: lack of time to counsel about DHSs

Pharmacists voiced that the limited time available for patient counselling surrounding over the counter (OTC) products obstructed proper pharmacist-patient communication [40, 42]. Due to time constraints, pharmacists were concerned about the extent of the information they could relay, as well as the time they required to investigate the suitability of various DHSs [33, 47]. As a result of limited time constraints, pharmacists tended to refer patients to alternative healthcare practitioners or answer patient queries briefly, as opposed to providing comprehensive information surrounding these products [42, 47].

#### Sub-theme 2.3: limited DHSs regulations

Pharmacists expressed their concerns regarding the limited DHS regulations and framework, which they had identified as a barrier when providing patients with information about such products [28, 30, 34, 35, 39, 41, 47]. In some countries, there are no requirements regarding whether a pharmacy should or should not sell DHSs [34]. Many pharmacists lacked knowledge about the regulations in their country of practice, which they had identified as a barrier to stocking DHSs in their pharmacy [33, 38]. Moreover, in contrast to traditional OTC pharmaceutical medications, limited regulations surrounding DHSs were a reason why pharmacists lacked the confidence to advise their patients about DHSs [32, 33, 41]. Similarly, these limited regulations lead some pharmacists to believe that these products are unsafe for use, and furthered their reluctance to recommend them to patients [48]. Furthermore, pharmacists suggested that regulations were lacking with respect to their role in dispensing DHSs, and called for regulatory and policy support [31, 39, 47].

### Theme 3: perceived role and importance of DHSs

#### Sub-theme 3.1: ambiguity in pharmacists’ professional roles

Due to a lack of education and training on DHSs, pharmacists were often unsure of their professional roles and responsibilities surrounding DHSs [33, 36, 40, 43, 47]. Some pharmacists understood that their role should involve providing adequate counselling, accurate information about products safety/efficacy, and recommending DHSs when appropriate [40, 47]. However, pharmacists’ lack of structured roles surrounding DHSs has affected their ability to assess the quality of counselling they provide to patients about these products [47]. Structure regarding how pharmacists should approach DHSs in their professional role is largely unknown, which makes it difficult for pharmacists to promote DHSs and advise patients on these products [40, 43, 47]. Pharmacists proposed that additional clarification on their role be provided to ensure a thorough understanding of their responsibilities pertaining to DHSs [40, 43, 47].

#### Sub-theme 3.2: perceived importance of DHSs

Many pharmacists perceived DHSs to be important [32, 38, 43, 45], and some even perceived DHSs to be as important as pharmaceutical medicine and vital to pharmacy practice [38, 43]. Some pharmacists suggested that their understanding of DHS importance stems from customer interactions, as they had expressed their desire for DHS use [45]. Furthermore, pharmacists have appeared to understand and empathize with customers’ demand for DHSs for their children, as they recognized that parents often seek alternative therapies for their children due to their own previous, negative experiences with pharmaceutical medications [45].

## Discussion

A total of 19 studies were deemed eligible and included in this review. An analysis of these studies identified the following themes: challenges to DHS education; post-graduation workplace challenges surrounding DHSs; and pharmacists’ perceived role and importance of DHSs. Findings from this review that may assist in improving pharmacists’ knowledge about DHSs and the safe usage of DHSs by patients is discussed below.

### Comparative literature

#### Challenges to DHS education

Findings across the literature support that pharmacists’ education related to DHSs was limited or non-existent. An American cross-sectional survey found that there were notable differences among pharmacy students’ recollection of when they had been introduced to DHSs in their program, despite taking the same courses [49]. This highlights the lack of structure surrounding DHSs in pharmacy curricula. This same study identified that the university did not have a course dedicated to DHSs, and suggested incorporating DHSs in didactic teaching [49]. Furthermore, a Jordanian cross-sectional study found that none of the pharmacists surveyed indicated that they had received any guidelines on DHSs during their formal training period before graduation [50].

The lack of DHSs education provided to pharmacists is likely responsible for their poor knowledge of these products, including drug-DHS interactions. An American survey found that only 2.4% of pharmacists who received inquiries surrounding DHSs felt that they could always answer the questions [51]. Moreover, a cross-sectional survey drew parallels between Saudi Arabian pharmacists and American pharmacists, in which both exhibited a lack of awareness of drug-DHS interactions [52]. Similarly, another American cross-sectional survey found that 26.3% of pharmacists provided inappropriate advice surrounding drug-DHS interactions [53]. This percentage of inaccurate drug-DHS advice is a reflection of pharmacists’ poor understanding of this topic. Notably, this lack of knowledge may be due to the near absence of premarket clinical trials of DHSs. One study found that patients with chronic disease were at the greatest risk of experiencing drug-DHS interactions, and it was suggested that part of this risk was due to the lack of premarketing regulations requiring safety trials on drug-DHS interactions [54]. Our finding suggest that a course dedicated to DHSs in pharmacy education may be of value, as well as improved regulations that mandate premarket clinical data for DHSs to test for drug-DHS interactions. Furthermore, the implementation of mandatory pharmacy practicums may be beneficial, as a Canadian assessment of pharmacy students’ knowledge suggested that fourth-year students who had completed a pharmacy practicum performed better on a standardized test about DHSs [55].

#### Post-graduation workplace challenges

There have been various calls for improved workplace training to maintain an adequate standard of DHSs knowledge among all pharmacists [18]. A systematic review identified that pharmacists agree that continuing education on DHSs should be mandatory, and noted that topics such as adverse drug reactions (ADRs), patient counselling, therapeutic uses and dosing would be most beneficial to include in this training [18]. Not only is workplace training on DHS limited, but DHSs-specific resources also appear to be lacking. For example, an American cross-sectional survey found that many pharmacists were not satisfied with the resources available to them [47, 56]. Therefore, one of the reasons pharmacists may be unable to counsel patients on the safe use of DHSs, is because  they lack the necessary information to do so. Another cross-sectional survey conducted in Saudi Arabia revealed that a major concern for pharmacists included a lack of scientific evidence that supported the use of DHSs [57]. To facilitate pharmacists’ acquisition of knowledge, DHS training and continuing education programs should be implemented; furthermore, they should have access to the most up-to-date clinical research findings surrounding DHSs.

Compounding this issue is the fact that pharmacists may lack the time to adequately counsel patients about DHSs. This finding is supported by a cross-sectional survey conducted in Saudi Arabia, which found that the most common barrier for pharmacists in providing DHS counselling to patients was a lack of time due to their other responsibilities [57]. Another study that interviewed pharmacists found that very few reported ADRs related to DHSs due to time constraints in the workplace [58]. Since a lack of time is an identified barrier to pharmacists counselling on DHSs or reporting DHS-related ADRs, the adjustment and re-evaluation of pharmacists’ assigned roles warrants further investigation.

The present review also identified that pharmacists were concerned over the limited regulations governing the sale of DHSs, which is another issue also supported by the peer-reviewed literature. A review of issues surrounding complementary and alternative medicine in the United States found that DHSs are not regulated nearly as strictly as pharmaceutical medications, specifically with respect to safety testing, efficacy, and marketing [59]. This is largely because, in the United States, the Food and Drug Administration does not require manufacturers to provide evidence that their DHSs are safe or effective before they are brought to market [18]. Additionally, an Australian survey examining barriers faced by pharmacists in providing complementary and alternative medicine information found that one of the main obstacles included a need for better DHSs regulations [60]. This same study specified that the need for better regulations included more rigorous standards for listing DHSs on the Australian Register of Therapeutic Goods, clearer information about the type of evaluation the DHS was subjected to, and improved labelling of warnings if product effectiveness was not established [60]. Interestingly, a cross-sectional survey found that American pharmacists were less confident in answering patient questions about DHSs when compared to health food store employees [21]. These feelings of hesitation and self-doubt might be attributed to the limited regulations on DHSs, specifically regarding the labelling. In 2015, the New York State attorney general’s office found that many DHSs sold by major retailers lacked the ingredients listed on the label or contained other substances not listed [61]. As a result, it is not unreasonable to infer that pharmacists working in community settings may have little confidence in the quality of such products. To restore pharmacists’ confidence in counselling about DHSs, policymakers, practitioners, and federal health agencies must collaborate to develop improved regulations that govern the sale, safety, and services of these products.

#### Pharmacists’ perceived role and importance of DHSs

The roles and responsibilities of pharmacists surrounding DHSs, along with standard DHS curricula and workplace knowledge requirements remain unclear across many jurisdictions [62]. This issue should be addressed and defined through clear regulations that define a pharmacist’s role and standards of knowledge surrounding DHSs should be included in pharmacy education so that trainees are aware of their future professional responsibilities. Despite the difficulties in acquiring DHS knowledge, pharmacists perceive such products to be important to their profession and their patients. An American study found that pharmacy students considered knowledge of DHSs to be very important [63]. Other studies have found that pharmacists viewed DHSs with importance and recognized the need for further continuing education and professional development opportunities surrounding DHSs [20, 40, 45].

#### Future directions for pharmacy education and training on DHSs

One future direction may include a collaboration between pharmacy associations and academic institutions, in order to encourage the development of guidelines that provide recommendations for DHSs curriculum, continuing education, and clinical practice. Based on the present review’s findings, it seems prudent that pharmacy students should be required to complete a course dedicated to the safety, efficacy, and regulations of DHSs within their professional training [49]. The Center for the Advancement of Pharmacy Education (CAPE) is an organization that has identified educational outcomes that should be considered when updating pharmacy curricula. CAPE has called for improved foundational knowledge and encouraged academic institutions to meet their suggested learning objectives in order to guide curricular revision and pharmacy programs [64]. To improve DHS knowledge and education through the re-evaluation and revision of pharmacy curricula, educational outcomes such as those provided by CAPE should be considered. Continuing professional development (CPD) programs such as those offered through the Canadian Pharmacists Association, specifically tailored towards DHSs may be useful in improving pharmacists’ knowledge of a variety of relevant topics, such as cannabis [65]. Finally, pharmacy educators need to be aware of the various web-based online resources and their quality, with respect to ADRs, dosing, and the side effects of DHSs, which have been previously summarized [66].

### Strengths and limitations

#### Common limitations identified by authors of eligible studies

Many included studies reported that a small sample size was identified as a limitation [31–33, 36, 40, 43, 45, 46]. The heterogeneity of the participants, with respect to factors such as age, sex, and educational background was also identified as a limitation across some included studies [40–42], as well as the fact that most participants were community pharmacists [30, 33–48]. The geographic distribution of participants was another limitation; some of the studies were conducted in jurisdictions that may not be representative of all pharmacists’ experiences country-wide [31, 38, 42, 43]. To some extent, this review has helped to mitigate this bias, as we combined data from all eligible studies to identify the commonalities between pharmacists working in different regions of the world. Additionally, all but one study incorporated an interview component into their study design [30–48]; reliance on interview data is subject to its own biases which include the researcher misinterpreting participants’ responses, or inadvertently influencing the participant to respond in a particular way. For example, if the interviewer is also a pharmacist, this may influence a participant’s answer differently than if the interviewer was not a healthcare professional [46, 47].

#### Strengths and limitations of the present systematic review

One strength of this study included a comprehensive search of the literature across multiple academic databases. Additionally, our systematic review followed PRISMA guidelines, and screening, data extraction and thematic analysis was conducted in duplicate and reviewed by a third author. One limitation includes the fact that only English language publications were considered for inclusion. Additionally, a small number of articles were irretrievable, despite receiving assistance from our university librarian in placing interlibrary loan requests. Lastly, it should be noted that reviews conducted on topics such as DHSs are limited by the operational definition selected.

## Conclusions

This review provides a summary of the barriers, knowledge, and training that pharmacists report related to counselling about DHSs. Our findings suggest that pharmacists lack general knowledge of DHSs, especially pertaining to drug-DHS interactions. Notably, this knowledge gap largely stems from limited pharmacy education and post-graduation training on DHSs, which decreased pharmacists’ confidence in providing counselling on DHSs and increased the ambiguity surrounding their professional role. Lack of reliable resources and time were among the most common barriers to counselling about DHSs. High-quality resources that provide safety and efficacy profiles of DHSs should be made easily accessible to pharmacists. Adjustment and re-evaluation of pharmacists’ assigned roles also warrants further investigation. Furthermore, there is a need to standardize the quantity and quality of DHS curriculum across pharmacy school programs, as well as workplace training and CPD programs surrounding DHSs. It is worthwhile to note that throughout this review, the topic of limited DHS regulations was frequently explored by the included studies and influenced many of the main findings, suggesting the need to further explore how the regulation of these products can be improved. Following the implementation of any of these recommendations, a future qualitative systematic review may serve useful in re-assess pharmacists’ experiences with respect to DHSs.

## Data Availability

All relevant data are included in this manuscript.

## Abbreviations

ADR: Adverse drug reaction
CAPE: Center for the Advancement of Pharmacy Education
CPD: Continuing professional development
DHS: Dietary and herbal supplement
OTC: Over-the-counter
PRISMA: Preferred Reporting Items for Systematic Reviews and Meta-Analyses
